# Clinical validation of circulating immune complexes for use as a diagnostic marker of canine leishmaniosis

**DOI:** 10.3389/fvets.2024.1368929

**Published:** 2024-03-18

**Authors:** Juliana Sarquis, Nuria Parody, Ana Montoya, Cristina Cacheiro-Llaguno, Juan Pedro Barrera, Rocío Checa, María Angeles Daza, Jerónimo Carnés, Guadalupe Miró

**Affiliations:** ^1^Department of Animal Health, Faculty of Veterinary, Universidad Complutense de Madrid, Madrid, Spain; ^2^R&D Unit Allergy and Immunology, LETI Pharma S.L.U., Madrid, Spain; ^3^Small Animal Emergency and ICU Service, Veterinary Teaching Hospital, Faculty of Veterinary, Universidad Complutense de Madrid, Madrid, Spain

**Keywords:** *Leishmania infantum*, immune complexes deposition, canine leishmaniosis, biomarker, PEG-ELISA

## Abstract

**Introduction:**

Canine leishmaniosis (CanL) is a systemic disease that affects dogs. When multiplication of the parasite cannot be controlled, dogs consistently show high levels of antigen and IgG antibodies, which lead to the formation of circulating immune complexes (CIC). Timely intervention to reduce the parasite load and CIC levels is crucial for preventing irreversible organ damage. However, a diagnostic test to quantify CIC levels is currently lacking.

**Methods:**

In this real-world study, we aimed to examine the performance of a new ELISA to measure CIC levels in dogs naturally infected with *Leishmania infantum*. Thirty-four dogs were treated according to their clinical condition and followed for 360 days. Before (day 0) and after treatment (days 30, 90, 180, 270, and 360), all dogs underwent a physical examination, and blood samples were obtained for CBC, biochemical profile, serum protein electrophoresis and IFAT. Serum PEG-precipitated CIC were determined by ELISA.

**Results:**

Our results indicate higher CIC levels in dogs in advanced disease stages showing higher antibody titres (*p* < 0.0001, *r* = 0.735), anemia (*p* < 0.0001), dysproteinemia (*p* < 0.0001), and proteinuria (*p* = 0.004). Importantly, dogs responding well to treatment exhibited declining CIC levels (*p* < 0.0001), while in poor responders and those experiencing relapses, CIC were consistently elevated. CIC emerged as a robust discriminator of relapse, with an area under the curve (AUC) of 0.808. The optimal cut-off to accurately identify relapse was an optical density of 1.539.

**Discussion:**

Our findings suggest that declining CIC levels should be expected in dogs showing a favorable treatment response. Conversely, in dogs displaying a poor response and recurrent clinical relapses, CIC levels will be high, emphasizing the need for vigilant monitoring. These findings suggest that CIC could serve as a valuable biomarker for disease progression, treatment efficacy, and relapse detection in CanL. Our study contributes to enhancing diagnostic approaches for CanL and underscores the potential of CIC as a complementary tool in veterinary practice. As we move forward, larger studies will be essential to confirm these findings and establish definitive cut-offs for clinical application.

## 1 Introduction

Canine leishmaniosis (CanL), a life-threatening zoonotic disease caused by an obligate intracellular protozoan *Leishmania infantum*, is transmitted to humans and other animals by blood-sucking phlebotomine sand flies. Dogs are considered the main peridomestic reservoir of infection (1, 2).

*Leishmania* may infect any organ, tissue or biological fluid, determining that clinical signs of CanL are non-specific and include lethargy, loss of appetite, weight loss, cutaneous lesions, onychogryphosis, generalized lymphadenomegaly, polyuria and polydipsia, ocular lesions, epistaxis, lameness, and other less common clinical signs like vomiting and diarrhea (3). However, many dogs show no apparent clinical signs or clinical-pathological abnormalities for several months or years after infection is confirmed. These dogs are defined as clinically healthy infected dogs. As for sick dogs, a classification system proposed by the LeishVet group helps guide practitioners in the clinical management of these animals and offers prognostic information (3). In this system, four clinical stages are described according to disease severity based on serological status, clinical signs, and laboratory findings.

After infection, clinically healthy infected and sick dogs show different immune responses to the parasite. Healthy dogs mount a robust Th1 response, which is crucial to halt parasite multiplication and for intracellular parasite elimination. In contrast, sick dogs mostly show a Th2 response, which leads to immune suppression and parasite proliferation (4, 5). In these animals, *Leishmania* antigens and non-specific IgG antibodies gradually build-up in the bloodstream as the parasite multiplies, leading to the formation of circulating immune complexes (CIC). These large molecules are deposited in the endothelium of several organs including the skin, eyes, joints, and renal glomeruli, and cause severe, often irreversible, organ damage. When this occurs, dogs can develop immune-mediated polyarthritis, vasculitis, indolent ulcers, and chronic uveitis, which can lead to blindness requiring the enucleation of one or both eyes. In LeishVet stages III or IV, CIC deposition in renal glomeruli can lead to chronic kidney disease (CKD) with life-threatening consequences (6–8).

The use of antimonials or miltefosine in combination with allopurinol is the standard treatment for CanL. In some cases, steroids are also needed to control immune-mediated clinical signs (9, 10). Prompt treatment is vital to reduce the parasite load and CIC levels, and thus mitigate the risk of irreversible organ damage. However, a diagnostic test to quantify CIC levels before and after treatment is currently lacking. Parody et al. (11) developed a method to quantify serum levels of CIC in dogs with CanL that was recently laboratory validated (12). In their work, Parody et al. (11) confirmed a clear association between disease progression and increasing CIC levels, suggesting that CIC could serve as a valuable biomarker for diagnostic purposes and also to track disease progression and assess treatment efficacy (13). Validating these findings in a real-world study is the first step toward making this method available to practitioners. The aim of the present study was to examine the performance of this new biomarker in a clinical setting by measuring CIC levels in dogs naturally infected with *L. infantum*.

## 2 Materials and methods

### 2.1 Study design

This longitudinal cohort study was conducted at the Infectious Diseases Unit of the Veterinary Teaching Hospital, Universidad Complutense de Madrid (UCM), Madrid, Spain. The study protocol was approved by this University’s Research Ethics Committee. Participants were dogs brought to the hospital by their owners over the period May 2021 to April 2022 for evaluation after a first diagnosis of CanL, or follow-up for check-ups or due to a clinical relapse. Dogs were considered eligible if naturally infected with *L. infantum* [positive serology for *Leishmania* by indirect fluorescent antibody test (IFAT >1:100, positive cytology and/or positive PCR result)] and their disease was then graded as LeishVet stages I–IV. Before enrolment, we informed all dog owners about the study protocol, including the option to withdraw their dogs from the study at any time, and obtained their written consent.

### 2.2 Study protocol

**Figure 1:**
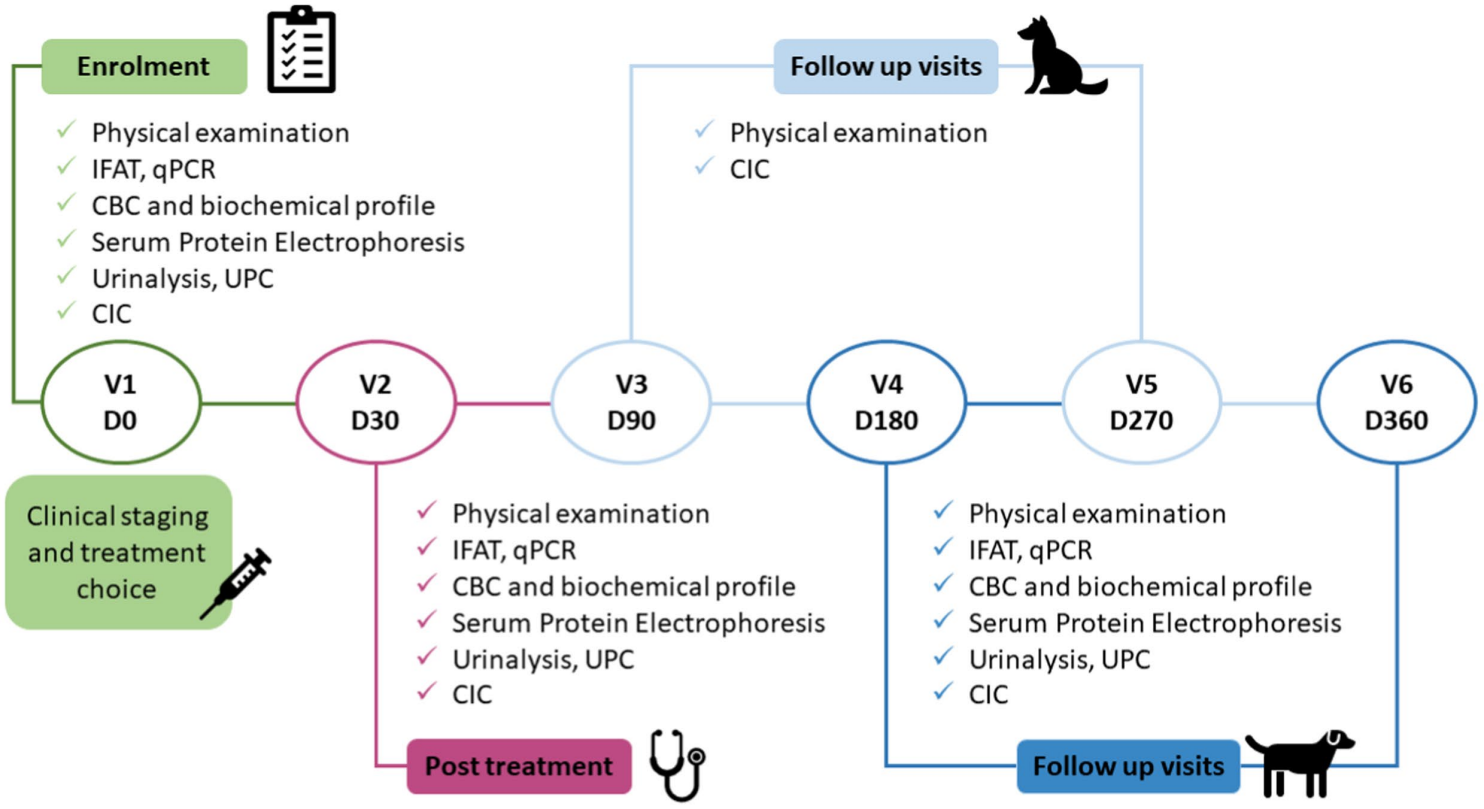
Study protocol. The clinical study consisted of six visits, enrolment (pre-treatment), after completing 30 days of treatment, and four follow-up visits. IFAT, indirect fluorescent antibody test; CBC, complete blood count; UPC, urinary protein/creatinine ratio; CIC, circulating immune complexes; qPCR, quantitative polymerase chain reaction.

Dogs were included in the study on day 0 (Visit 1 enrolment pre-treatment) and thereafter followed for 12 months. Appropriate treatment was started shortly after enrolment. Visits to our service were then scheduled for post-treatment onset days 30, 90, 180, 270, and 360 (Figure 1).

**Figure 2:**
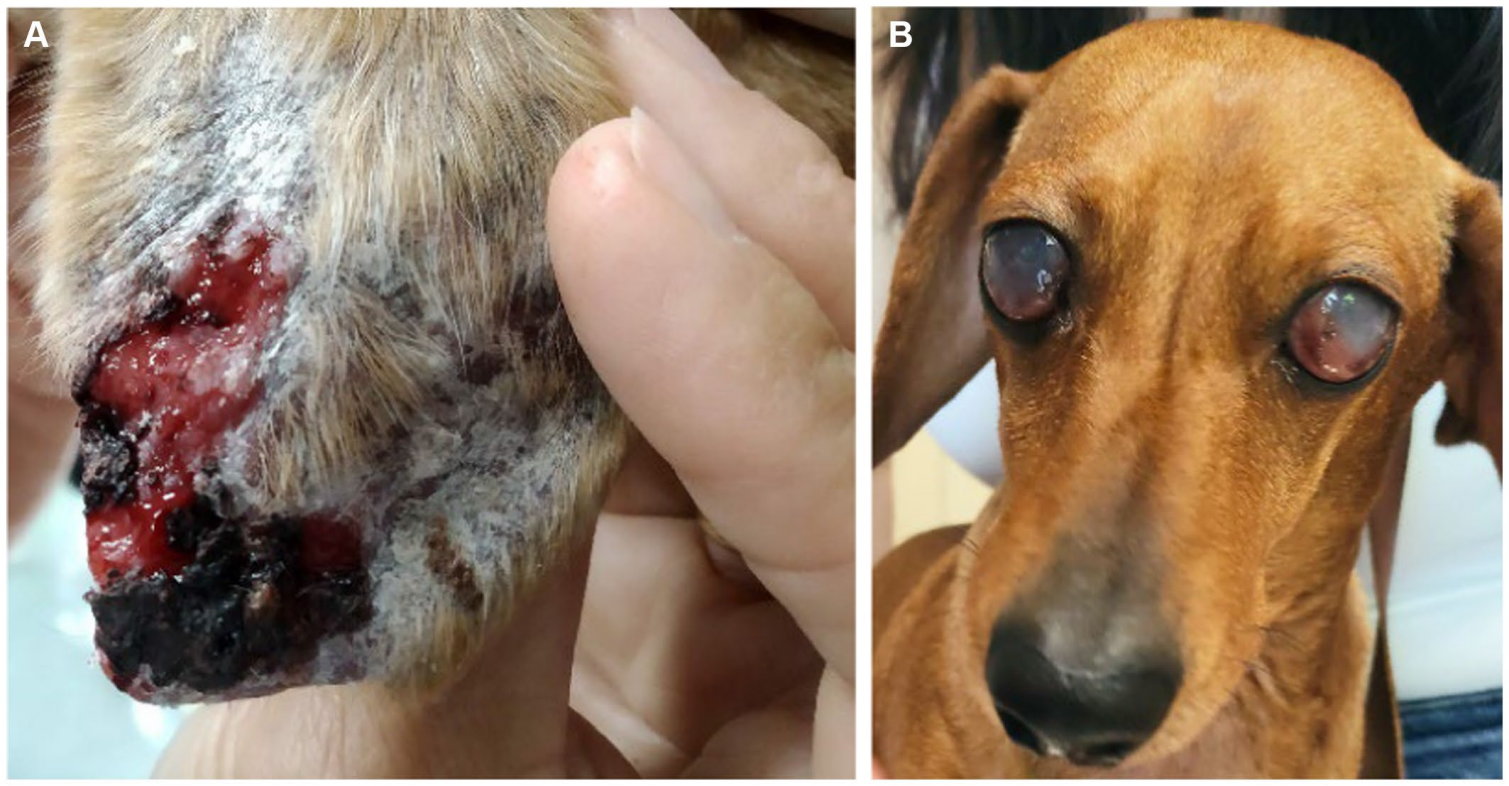
Clinical signs of CIC deposition in dogs with CanL. **(A)** Ulcer in the pinna associated with vasculitis. **(B)** Blindness associated with bilateral uveitis.

Dogs underwent a thorough physical examination in each visit. Data were compiled on clinical signs including signs of CIC deposition (Figure 2). Each clinical sign was graded from 0 to 3 (low to high severity) to give an overall clinical score for each animal (maximum 68) (Table 1).

**Table 1:** Clinical scoring system used in this study.

Clinical signs	Score			
	0	1	2	3
Apathy	Absent	Mild (unable to properly run)	Severe (intolerant to exercise)	Prostration (refuses to walk)
Appetite	Normal	Slightly reduced (> half normal intake)	Markedly reduced (< half normal intake)	Anorexia
Weight loss	Absent	Mild (<10%)	Moderate (10-20%)	Severe (>20%)
Fever	Absent	Temperature 1°C above normal	Temperature 2°C above normal	Temperature ≥ 3°C above normal
Pale mucous membranes	Absent	Mild	Moderate	Severe (white mucous membranes)
Adenopathy	Absent	1 or 2 enlarged lymph nodes	More than 2 enlarged lymph nodes	Generalized lymphadenomegaly
Splenomegaly	Absent	–	Present	–
Oral ulcers	Absent	Small focal ulcers	Multiple medium-size ulcers	Multiple oral ulcers, mastication impaired
Onychogryphosis	Absent	–	Present	–
Seborrhoeic dermatitis	Absent	<10% body surface	<25% body surface	Generalized seborrhoeic dermatitis
Hyperkeratosis	Absent	<10% body surface	<25% body surface	>25% body surface
Conjunctivitis	Absent	Mild unilateral	Severe unilateral or mild or moderate bilateral	Severe bilateral
Uveitis	Absent	Mild, resolved with topical treatment	Moderate, requiring oral treatment	Severe risk of blindness
Epistaxis	Absent	Occasional bleeding	Frequent bleeding	Uncontrolled bleeding
Vasculitis	Absent	Mild lesions	Moderate lesions	Severe lesions
Lameness	Absent	Mild (no impact on movement)	Moderate lameness (mild pain or discomfort)	Severe lameness (movement impaired)
Ulcers	Absent	Small focal ulcers	Multiple medium-sized ulcers	Severe ulcerated lesions
Polyuria	Absent	–	Present	–
Polydipsia	Absent	–	Present	–
Haematuria	Absent	Blood on sediment analysis	Visible hematuria	Red urine
Dysuria	Absent	–	Present	–
Urinary incontinence	Absent	–	Present	–
Vomiting	Absent	Occasional	Frequent	Vomit bloody
Diarrhea	Absent	Occasional	Frequent	Diarrhea with blood/melena

Adapted from Miró et al. (14).

At each visit, blood samples were collected to determine serum CIC concentrations by PEG ELISA (11) (Figure 1). On days 0, 30, 180, and 360, routine laboratory tests were also performed to determine the clinical condition of the dog, which included complete blood count (CBC), biochemical profile (creatinine, urea, and alanine aminotransferase), serum protein electrophoresis, and anti-*Leishmania* immunofluorescence antibody test (IFAT) (15). Serial dilutions from 1:50 to 1:6400 were used and seropositivity defined by a cut-off ≥1:200. Urinalysis, including urine density, sediment analysis and protein/creatinine ratio (UPC), were also undertaken on days 0, 30, 180, and 360 on urine samples obtained preferably by cystocentesis, or by free catch into a sterile container. Quantitative PCR was also performed to confirm the infection on day 0, and to quantify parasite load at 6 and 12 months post enrolment. Based on physical examination and clinical-pathological findings, dogs were assigned to LeishVet clinical disease stages I–IV (3).

### 2.3 CIC isolation and quantification

A modified precipitation method with polyethylene glycol (PEG; Sigma-Aldrich, St. Louis, MO, United States) was used to separate free antigens and antibodies from CIC (11). PEG-precipitated CIC were pelleted by centrifugation, then reconstituted in 0.01 M phosphate-buffered saline (PBS) and stored at −80°C for further use. CIC were quantified using a *Leishmania*-specific ELISA method as described by Parody et al. (11). All samples were tested in duplicate, and the mean value was recorded. A mean OD of 0.274 + 3 standard deviations of negative samples was considered as the cut-off value; any sample exhibiting absorbance above the cut-off value was considered positive.

### 2.4 Treatment

The treatment for each dog was determined by one of the authors (a veterinarian specialist in infectious diseases) and was based on clinical history, physical examination, and laboratory findings. Clinically healthy infected dogs or those at stage I received no treatment or received only allopurinol for 6–12 months. Dogs in stages II or III were treated with a combination of meglumine antimoniate (MGA) (50 mg/kg subcutaneously q12h for 28 days) and allopurinol (10 mg/kg orally q12h for 6–12 months). When considered necessary, prednisone (0.5 mg/kg orally q12h for 3–4 weeks) was added to the treatment regimen in dogs in stage III to control signs of CIC deposition (10). Proteinuric dogs were treated following International Renal Interest Society Guidelines for CKD in dogs (16).

Relapses were also recorded and a second course of leishmanicide was administered if needed. Disease relapse was defined as significant clinical worsening and/or laboratory abnormalities prompting a new course of treatment with antimonials.

The clinical response to treatment was assessed by examining clinical records, changes produced in clinical scores and laboratory variables over time, and the number of relapses produced during the study period. Good responders were dogs that showed improvement in clinical signs and laboratory abnormalities and no relapses in the 12 months following treatment onset. Dogs were considered poor responders if they experienced one or more relapses during the study or showed no improvement in clinical-pathological variables by the end of the study.

### 2.5 Statistical analysis

All statistical tests were performed using the software packages SAS (SAS Institute Inc., Cary, NC, United States) version 9.4, and SPSS Statistics version 28 (SPSS Inc., Chicago, IL, United States). Graphs were created using GraphPad Prism 10. Significance was set at *p* ≤ 0.05.

Some variables were categorized for analysis: age (2–4 years: young adult, 5–8 years: mature adult, ≥9 years: senior) (17), IFAT titer according to cut-off values established by the laboratory (<200 negative, 200–400 low positive, 800–1,600 medium positive, >1,600 high positive) (3, 15, 18, 19), and albumin/globulin ratio (A/G) (>0.8 normal, 0.7–0.8 mild dysproteinemia, 0.5–0.69 moderate dysproteinemia, <0.5 severe dysproteinemia) (19–21).

As CIC values within groups were not normally distributed (Kolmogorov–Smirnov test, *p* < 0.001), non-parametric tests were used. The Wilcoxon test was used to compare categorical variables between two established groups and the Kruskal–Wallis test with Bonferroni correction when there were more than two groups. The association between CIC levels and the clinical signs from the scoring system used in this study was evaluated using the Chi-Square test, specifically analyzing the 2 × 2 contingency table formed by the variables. From this analysis, relative risks (RR) for each clinical sign were calculated, accompanied by their respective 95% confidence intervals (CI). These measures serve to quantify the strength and direction of the association between CIC and the clinical signs, providing valuable insights into their relationship.

To assess changes in CIC levels over time, we used the Friedman test for repeated measures also with Bonferroni correction. Spearman’s correlation test was used to examine possible correlations between CIC levels and continuous variables. We also assessed the capacity of CIC level to accurately identify relapse by calculating the area under the receiver operating characteristic (ROC) curve (AUC).

## 3 Results

### 3.1 Study population

Of the 44 dogs enrolled, 23 were male and 21 female. Fourteen dogs were classified as young adults (2–4 years), 18 as mature adults (5–8 years), and 11 as senior (mean age 6.6 years; 95% CI: 1.6–14.64). During the study, three dogs died of a comorbidity (2 lymphoma, 1 carcinoma), and seven were lost to follow-up as owners failed to return for the re-visits. Even though only 34 dogs completed the 12 months of the study, data for all 44 dogs enrolled were included in the analysis until the loss of follow-up.

The CIC levels were significantly higher in young adult dogs compared to senior (*p* < 0.001), and low negative correlation was found between CIC level and age (*r* = −0.273). No differences in CIC levels were detected between mongrels and pure breeds (*p* = 0.107) or between male and female dogs (*p* = 0.416).

### 3.2 CIC and clinical scores, and LeishVet stages

**Figure 3:**
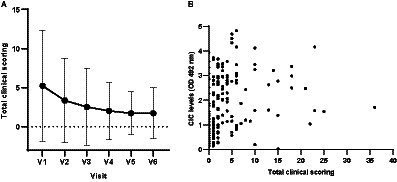
**(A)** Means and SDs of total clinical scores recorded in each visit. **(B)** Correlation between total clinical scores and CIC levels (Spearman correlation, *r* = 0.5135). V1 (Day 0), V2 (Day 30), V3 (Day 90), V4 (Day 180), V5 (Day 270), and V6 (Day 360).

The mean clinical score awarded to the 34 dogs that completed the 12 months of follow up gradually decreased throughout the study (Figure 3), and clinical scores and CIC levels showed positive correlation (Figure 3) (*r* = 0.5135, *p* < 0.0001). Dogs showing clinical signs were also found to have a greater risk of having elevated CIC levels. Table 2 displays the relative risks (RR) for various clinical signs evaluated in association with the presence of CIC in dogs. Interestingly, 10/44 dogs (22.72%) had a clinical score of five or less, but high CIC levels (≥2 OD).

**Table 2:** Relative risk of clinical signs associated with a significant risk of elevated circulating immune complexes (CIC).

Clinical sign		Elevated CIC		Relative risk [95% CI]	*p*-value
		Yes	No		
Apathy	Yes	17	1	2.58 [2.08–3.20]	<0.0001
	No	72	125		
Weight loss	Yes	12	7	1.60 [1.09–2.36]	0.0437
	No	77	119		
Adenopathy	Yes	43	13	2.65 [1.99–3.52]	<0.0001
	No	46	113		
Pale mucous membranes	Yes	20	2	2.54 [2.01–3.20]	<0.0001
	No	69	124		
Uveitis	Yes	20	3	2.41 [1.89–3.09]	<0.0001
	No	69	123		
Lameness	Yes	26	9	2.12 [1.60–2.80]	<0.0001
	No	63	117		
Hyperkeratosis	Yes	20	7	2.01 [1.50–2.70]	0.0002
	No	69	119		
Seborrhoeic dermatitis	Yes	20	11	1.72 [1.24–2.37]	0.0047
	No	69	115		
Ulcers	Yes	15	8	1.69 [1.20–2.39]	0.0141
	No	74	118		

Repeated measures were treated as independent measures for relative risk calculation. CI, confidence interval.

**Figure 4:**
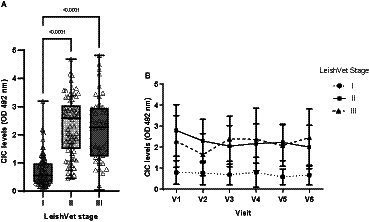
**(A)** CIC levels recorded in the dogs assigned to different LeishVet stages (non-parametric Kruskal–Wallis test). **(B)** Changes in mean CIC levels recorded over the 12-month study period in dogs at different LeishVet stages. V1 (Day 0), V2 (Day 30), V3 (Day 90), V4 (Day 180), V5 (Day 270), and V6 (Day 360).

At enrolment, 15 dogs were classified as LeishVet stage I, 20 as stage II, 7 as stage III and 2 as stage IV. Owing to the small number of animals at stage IV, data for stages III and IV were combined for analysis. Dogs at stage I showed significantly lower CIC levels than those classified as having CanL stages II and III (*p* < 0.0001) (Figure 4). No differences were found between dogs staged as II or III (*p* = 0.485). Moreover, stage I dogs consistently maintained low CIC levels throughout the study (Figure 4). In stage II patients, CIC levels decreased after treatment and remained stable, whereas in stage III patients, they increased after 90 and 360 days of treatment (Figure 4).

### 3.3 CIC and laboratory findings

**Figure 5:**
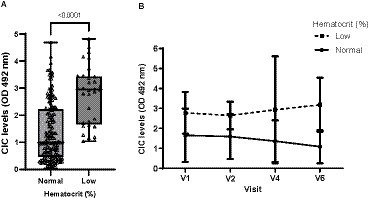
**(A)** CIC levels recorded in dogs with or without anemia (non-parametric Wilcoxon test). **(B)** Changes in CIC means produced during the study in dogs with or without anemia. Reference value hematocrit: 37–55%. V1 (Day 0), V2 (Day 30), V3 (Day 90), V4 (Day 180), V5 (Day 270), and V6 (Day 360).

Leukopenia was the most frequent abnormality detected in the CBC, occurring in 27.78% of dogs, followed by thrombocytopenia (22.62%) and non-regenerative anemia (18.23%). However, significant differences in CIC levels were only found for anemic dogs (*p* < 0.0001) (Figure 5), which not only had higher CIC levels but also experienced a gradual increase in these levels as early as 6 months after treatment cessation (Figure 5). Finally, we observed moderate negative correlation between hematocrit and CIC level (*r* = −0.547).

Among the biochemical variables measured, creatinine was found elevated in only 8.57% of dogs, whereas alanine aminotransferase (ALT) was increased in 11.49%. No significant differences were found in CIC levels between dogs with normal or elevated creatinine (*p* = 0.789, *r* = −0.327) or ALT (*p* = 0.434, *r* = −0.216) levels.

**Figure 6:**
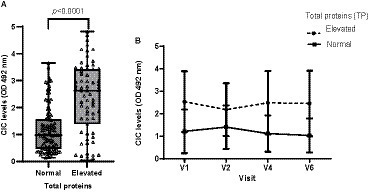
**(A)** CIC levels recorded in dogs with or without hyperproteinemia (non-parametric Wilcoxon test). **(B)** Changes in mean CIC levels produced during the study in dogs with or without hyperproteinemia. Normal reference value total proteins: 5,8–7,5 g/dL. Frequencies: Normal: 57.78%; hyperproteinemia: 37.22%. V1 (Day 0), V2 (Day 30), V4 (Day 180), and V6 (Day 360).

Hyperproteinemia was detected in 37.22% of visits. Dogs displaying hyperproteinemia had significantly higher CIC levels (*p* < 0.0001) (Figure 6). At visit 3, which was the first follow up visit after the end of treatment, both groups showed reduced CIC levels. However, in dogs with dysproteinemia, these levels subsequently increased, while dogs without dysproteinemia consistently maintained lower CIC levels (Figure 6). Additionally, we found moderate positive correlation between CIC and total protein levels (*r* = 0.533, *p* < 0.0001).

**Figure 7:**
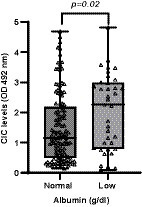
CIC levels recorded in dogs according to albumin level (non-parametric Wilcoxon test). Normal reference value albumin: 2.4–3.9 g/dL.

Hypoalbuminemia was present in 22.02% of patients. Dogs with hypoalbuminemia displayed significantly more elevated CIC levels (*p* = 0.02) (Figure 7), and low negative correlation was found between CIC and albumin levels (*r* = −0.306).

**Figure 8:**
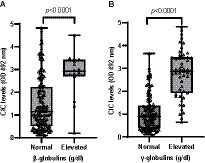
**(A)** CIC levels recorded in dogs according to the presence of beta-or **(B)** gamma-hyperglobulinemia (non-parametric Wilcoxon test). Normal reference values beta-globulins: 1.3–2.7 g/dL; gamma-globulins: 0.5–2 g/dL.

Among the globulins, gamma globulin was the most affected with hypergammaglobulinemia present in 31.1% of animals, followed by hyperbetaglobulinemia (9.82%). Dogs with these abnormalities showed significantly higher CIC levels (*p* < 0.0001) (Figure 8), and we found positive correlation between CIC and beta (*r* = 0.444) and gamma (*r* = 0.720) globulin levels. Elevated alpha-1 and alpha-2 globulins were noted in 2.48 and 8.18% of dogs, respectively, but no differences in CIC levels were detected between dogs with normal or high blood alpha-1 globulins (*p* = 0.960). Dogs with elevated alpha-2 globulins had higher CIC levels (*p* = 0.022). No correlation was found between CIC levels and alpha-1 and alpha-2 globulins (*r* = 0.021; *r* = −0.022, respectively).

**Figure 9:**
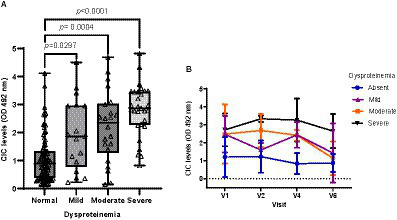
**(A)** CIC levels in dogs with a normal *A*/*G* ratio and different degrees of dysproteinemia (non-parametric Kruskal–Wallis test). **(B)** Changes in CIC means detected in dogs with a normal *A*/*G* ratio and different degrees of dysproteinemia. Normal *A*/*G* ratio > 0.8; mild dysproteinemia: 0.7–0.8; moderate dysproteinemia: 0.6–0.5; severe dysproteinemia: *A*/*G* < 0.5. V1 (Day 0), V2 (Day 30), V4 (Day 180), and V6 (Day 360).

Among all variables examined, dysproteinemia was the most common laboratory finding, detected in 41.66% of animals. Dogs with dysproteinemia showed significantly higher CIC levels (*p* < 0.0001), and high negative correlation was found between CIC level and the A/G ratio (*r* = −0.618). When we assessed the effect of the level of dysproteinemia on CIC level (Figure 9), we found no differences between dogs with mild (*p* = 1.0), moderate (*p* = 0.419) or severe (*p* = 0.121) dysproteinemia. Throughout the study, CIC levels remained below 1.5 OD in dogs without dysproteinemia, while levels were consistently over 2.5 OD in those with severe dysproteinemia (Figure 9).

A large proportion (36.18%) of dogs showed a normal UPC ratio. Borderline proteinuria and proteinuria were recorded in 26.32 and 36.18% of the patients, respectively. Dogs with borderline proteinuria (UPC = 0.2–0.5) had higher CIC levels than dogs without proteinuria (UPC < 0.5) (*p* = 0.035, *r* = 0.172). However, no differences in CIC were found between dogs with borderline proteinuria or proteinuria (*p* = 0.69).

### 3.4 CIC and IFAT titres

**Figure 10:**
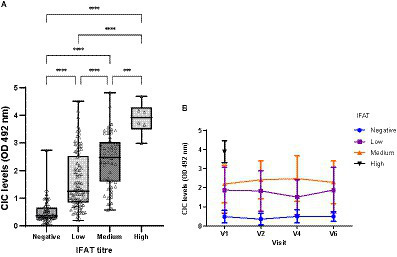
**(A)** CIC levels recorded in dogs showing different titres of antibodies against *L. infantum*. IFAT < 200 negative, 200–400 low, 800–1600 medium, >1600 high (non-parametric Kruskal–Wallis test; ^***^p < 0.0005, ^****^p < 0.0001). **(B)** Changes in CIC means produced during the study in dogs showing different antibody titres against *L. infantum*. High titres are not displayed due to the low number detected. V1 (Day 0), V2 (Day 30), V4 (Day 180), and V6 (Day 360).

The IFAT titres ranged from negative to 1:6400, with the cut-off set at >1/100. Significant differences in CIC levels were observed among groups showing negative, low, medium, or high antibody titres (*p* < 0.0001) (Figure 10), and there was strong positive correlation between CIC levels and IFAT titres (*r* = 0.735; *p* < 0.0001). Further, throughout the study, CIC levels remained consistently low in clinically healthy infected dogs returning negative IFAT results (Figure 10).

### 3.5 CIC and treatment

As CanL is a chronic disease, at the time of enrolment some dogs were already receiving treatment with allopurinol (*n* = 15/44), domperidone (*n* = 1/44) (22–24), nucleotides (*n* = 5/44) (25–28), prednisone (*n* = 1/44) or the angiotensin-converting enzyme inhibitor benazepril (*n* = 2/44). The treatment regimens of the dogs after enrolment in this study are detailed in Table 3.

**Table 3:** Treatment regimens of dogs included in this study.

Treatment post-enrolment		Number of dogs
Meglumine antimoniate + allopurinol *n* = 23/44 (lost = 3)	+ prednisone	4/23
	+ nucleotides^1^	7/23
Allopurinol *n* = 11/44 (lost = 4)	+ prednisone	1/11
	+ nucleotides^1^	4/11
	+ domperidone^2^	1/11
Nucleotides *n* = 2 (lost = 1)		2/44
No treatment *n* = 8 (lost = 2)		8/44

^1^Impromune^®^ (Bioiberica). ^2^Leisguard^®^ (Ecuphar).

The CIC levels were significantly higher in dogs treated with meglumine antimoniate (*p* = 0.0036) or allopurinol (*p* = 0.015) and in those with proteinuria treated with benazepril (*p* = 0.0039). Dogs treated with meglumine antimoniate or allopurinol or that received benazepril to control proteinuria were almost two times more likely to have high CIC levels (Table 4). In contrast, no differences were detected according to treatment with nucleotides (*p* = 0.0519) or prednisone (*p* = 0.2312).

**Table 4:** Relative risk of treatment regimens associated with a significant risk of elevated circulating immune complexes (CIC).

Treatment		Elevated CIC		Relative risk [95% CI]	*p*-value
		Yes	No		
MGA	Yes	16	6	1.90 [1.39–2.60]	0.0018
	No	74	120		
Allopurinol	Yes	64	72	1.44 [1.00–2.08]	0.0361
	No	26	54		
Benazepril	Yes	14	6	1.80 [1.28–2.52]	0.007
	No	76	120		

Repeated measures were treated as independent measures for relative risk calculation. MGA, meglumine antimoniate; CI, confidence interval.

### 3.6 CIC and treatment response

**Figure 11:**
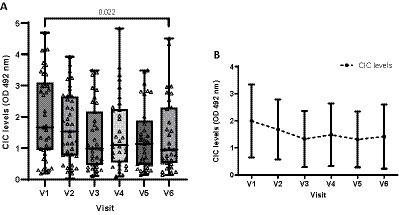
Box chart **(A)** and **(B)** line graph displaying changes in mean CIC levels recorded during the study. **(A)** Non-parametric Friedman test.

To assess the impacts of treatment in sick dogs, we also examined CIC levels over time. As the data were not normally distributed, we employed the non-parametric Friedman test. Nine of the 44 dogs, each missing one visit, were excluded from this part of the analysis. Our results show that mean CIC levels gradually decreased after treatment and remained relatively stable throughout the study period (Figure 11). Nevertheless, significant differences in CIC levels were only detected between the first (D0) and last visit (D360) (Friedman test *p* = 0.022) (Figure 11).

**Figure 12:**
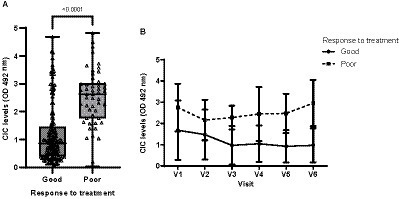
**(A)** CIC levels recorded in dogs showing a good or poor response to treatment (non-parametric Wilcoxon test). **(B)** Changes in CIC means produced during the study in dogs showing a good or poor response to treatment. V1 (Day 0), V2 (Day 30), V3 (Day 90), V4 (Day 180), V5 (Day 270), and V6 (Day 360).

Of the 44 dogs initially included in our study, 28 were classified as good responders, 11 as poor responders and 5 could not be classified, as they were lost to follow-up. Good responders showed significantly lower CIC levels than poor responders (*p* < 0.0001) (Figure 12). Moreover, good responders showed lower CIC levels and they declined throughout the study (Figure 12). In poor responders, while CIC reduction was observed after treatment, levels progressively increased as early as 90 days later (Figure 12).

### 3.7 CIC and relapses

Relapses were detected in 23.18% of the animals. At inclusion, 18 of the 44 dogs had had a previous diagnosis of CanL and were referred for treatment due to clinical relapse. Over the 12-month follow-up period, six dogs had one relapse and two had two relapses (Table 5).

**Table 5:** Number of relapses detected during the study and their time points.

Dog ID	No of relapses	Time-point	Treatment
01	2	V4 (D180), V6 (D360)	MGA and allopurinol
04	1	V6 (D360)	MGA and allopurinol
05	2	V4 (D180), V6 (D360)	MGA and allopurinol
06	1	V6 (D360)	MGA and allopurinol
20	1	V4 (D180)	MGA and allopurinol
29	1	V6 (D360)	MGA and allopurinol
32	1	V5 (D270)	Initiation of allopurinol
33	1	V5 (D270)	MGA and allopurinol

MGA, meglumine antimoniate.

**Figure 13:**
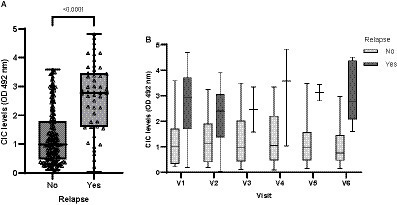
**(A)** CIC levels recorded in dogs experiencing or not experiencing relapse (non-parametric Wilcoxon test). **(B)** CIC levels recorded over the study period in dogs experiencing or not experiencing relapse. V1 (Day 0), V2 (Day 30), V3 (Day 90), V4 (Day 180), V5 (Day 270), and V6 (Day 360).

Dogs experiencing relapse had significantly higher CIC levels compared to dogs not experiencing relapse (*p* < 0.0001) (Figure 13); these were elevated consistently in all visits (OD > 2) (Figure 13).

**Figure 14:**
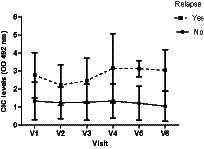
Changes in CIC means recorded over the study in dogs experiencing or not experiencing relapse. V1 (Day 0), V2 (Day 30), V3 (Day 90), V4 (Day 180), V5 (Day 270), and V6 (Day 360).

Interestingly, we detected markedly increased CIC levels in V4 (D180) (Figure 14), persisting into V5 (D270) and V6 (D360) and coinciding with the relapses recorded in these dogs (Table 5). In contrast, dogs not relapsing had CIC levels below 2 OD during the entire study period (Figure 14).

**Figure 15:**
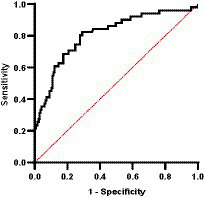
ROC curve analysis of relapse (AUC = 0.8085, *p* < 0.0001).

Importantly, through ROC curve analysis we were able to determine that CIC was a good discriminator of relapse (AUC = 0.808, *p* < 0.0001; 95% CI: 0.736–0.881) (Figure 15). Our results indicate that the optimal cut-off to correctly identify a relapse was 1.539 OD (sensitivity 82.4%; specificity 70.9%).

## 4 Discussion

To our knowledge, this is the first study to clinically validate the use of CICs as biomarkers of the progression of canine leishmaniosis. Dogs in advanced stages of this disease show higher CIC levels (11) and a higher risk of multiple organ damage, which can significantly impair quality of life and dramatically reduce survival (6–8, 29). Currently, diagnostic methods for quantifying CIC are lacking. Parody et al. (11) introduced a non-invasive *Leishmania*-specific method of measuring CIC levels in serum samples from dogs with leishmaniosis. This method was recently laboratory validated following the recommendations of the NIH Biomarkers group (12). The method’s specificity and robustness suggest this new biomarker could be useful not only for a diagnosis of CanL but also for tracking disease progression and potentially assessing treatment efficacy (13). In the present real-world study, we sought to clinically validate the performance of this new biomarker.

In our study, 34 dogs naturally infected with *L. infantum* at different LeishVet stages were followed for 12 months. Our data show that CIC levels are significantly higher in young adult dogs than senior dogs (*p* < 0.001) while no differences were detected according to sex or breed. The influence of sex, breed, and age on susceptibility to acquiring the infection has been explored in numerous studies with contrasting results (30–34). Nonetheless, the authors of two epidemiologic studies involving a large number of dogs, which took into account the presence of clinical signs of CanL, described that the age distribution for affected animals was bimodal, with a first peak corresponding to animals of around 3 years of age and a second, less evident, peak representing animals around 8 years old (30, 31). Age also seems to be an important risk factor associated with visceral leishmaniasis (VL) in humans according to two systematic reviews (35, 36) such that children are more susceptible to both infection and illness. Our results point to younger dogs controlling the disease less effectively and thus being more likely to have formed CIC.

Mean clinical scores progressively declined during the study and significant differences were detected in total clinical scores between visits. Moreover, we found positive correlation between clinical score and CIC levels (*r* = 0.5135, *p* < 0.0001). These results suggest that a decrease in clinical score will be accompanied by a drop in CIC levels. Nevertheless, some dogs had a low clinical score (≤5) despite having high levels of CIC (≥2 OD). The only clinical signs observed in these dogs were lameness, uveitis, skin ulcers, or polyuria/polydipsia. Hence, measuring CIC levels could be useful in dogs that do not have an overall picture of generalized CanL yet show clinical signs of immune complex deposition. Future studies will bring light to the relationship between clinical scores and CIC levels.

The CIC levels were significantly lower in stage I dogs compared to stages II and III, consistent with the findings of Parody et al. (11), who reported a clear association between CIC levels and disease progression. In our study, we examined trends in CIC levels over 12 months in dogs at different clinical stages of CanL. Our results indicate that dogs in early disease stages have low CIC levels that remain low for long periods. Conversely, in advanced stages, dogs consistently show high levels of immune complexes, and increases may be observed as early as 90 days after initiating specific treatment. These results highlight the importance of close and frequent monitoring of CIC deposition in dogs in advanced stages of CanL, and suggest this new biomarker could be used to detect relapses.

Non-regenerative anemia is one of the most frequent clinical-pathological abnormalities reported in dogs with CanL. Some authors describe correlation between anemia or the severity of clinical signs (21, 37, 38) and parasite load (38–40). Dysproteinemia is also frequently observed in dogs with CanL (19–21). Protein electrophoresis has proven extremely useful for the diagnosis and monitoring of CanL and is routinely performed in clinical practice (41–44). Indeed, several studies have shown that this technique may show abnormalities very early during the course of disease, even before the onset of overt clinical signs (21, 41, 42, 45, 46). In our study, we observed strong correlation between CIC and hematocrit (*r* = 0.524), total proteins (*r* = 0.507), beta- (*r* = 0.426) and gamma globulins (*r* = 0.673), and the *A*/*G* ratio (*r* = −0.647). Further, our results also show that dogs with non-regenerative anemia and/or dysproteinemia have significantly higher CIC levels. When we examined the kinetics of these immune complexes, we noted that dogs with anemia and/or dysproteinemia showed higher CIC levels throughout the study despite specific treatment. Taken together, our findings point to the high reliability of this new biomarker, as it correlates with other tools used for the diagnosis and clinical management of CanL. Moreover, they confirm that dogs with anemia or dysproteinemia are at a higher risk of CIC deposition in specific organs.

The IFAT is one of the most used quantitative serologic techniques for the detection of anti-*Leishmania* antibodies and is considered the gold standard test for *Leishmania* infection. Extensive research has identified correlation between IFAT results and clinical signs (47–51), becoming more evident in severe clinical forms of CanL (51). In human VL, high anti-*Leishmania* antibodies also correlate with disease progression (4). Studies have also shown that IgG antibodies not only fail to protect against this intracellular parasite but also contribute to disease progression by reversing the inflammatory cytokine profile of immune cells and inducing the production of high IL-10 levels via the receptor FcγRIII (4, 52). Here, significant differences in CIC levels were found among dogs showing negative, low, medium or high antibody titres, and high positive correlation was observed between CIC and IFAT results (*r* = 0.735). These findings are in line with those described by Parody et al. (11), who also noted high correlation between these two variables (*r* = 0.754). Thus, dogs with high antibody titres have significantly higher CIC levels and are thus at greater risk of their deposition in target organs.

Mean CIC levels dropped after treatment and remained under 1.5 OD throughout the study, although significant differences were only found between the first and the last visits. Likely reasons for this are: (1) the small number of animals included in the study, and thus reduced statistical power when using the Friedman test with Bonferroni correction; and (2) in dogs showing a poor response to treatment, CIC levels can remain elevated for several months. In this study, sick dogs were treated with antimonials in combination with allopurinol, which is the standard treatment for CanL in Europe (3). Four dogs treated with antimonials, and one treated with allopurinol also received prednisone for 1 month to control clinical signs of CIC deposition. Dogs treated with antimonials or allopurinol, or those that received benazepril to control proteinuria, had significantly higher CIC levels and were up to two times more likely to have elevated CIC levels in serum. This is not surprising as, except for allopurinol, these medications are used in more advanced stages of the disease. Due to the small number of animals that received treatment with prednisone, no conclusions can be made regarding the kinetics of CIC levels in these dogs. Further studies are needed to examine the optimal use of this new biomarker in tailoring and monitoring corticosteroid use in dogs experiencing CIC deposition.

Good responders had significantly lower CIC levels compared to poor responders (*p* < 0.0001), which progressively decreased over the study course. In poor responders, although a CIC reduction was observed after treatment, levels progressively increased as early as 90 days post-treatment. These results suggest that measuring CIC levels before and after treatment could serve to assess treatment effectiveness.

Dogs experiencing relapse after treatment had significantly higher CIC levels (*p* < 0.0001); over 2 OD in all visits. During the study, six dogs had one relapse and two had two relapses. Most relapses occurred in visit 6, 1 year after treatment. Nevertheless, three dogs had a relapse as early as at 6 months post-treatment. Studies have shown that the presence of immune complexes contributes to the establishment of chronic infections in murine models and human VL (4, 52). One key mechanism is their ability to stimulate macrophages and other immune cells to produce IL10. This cytokine reduces the expression of inducible nitric oxide synthase (iNOS) and the intracellular production of nitric oxide, which are both vital for eliminating intracellular *Leishmania* parasites. Moreover, IL-10 downregulates the Th1-associated IFN-γ response, which is essential to activate nitric oxide production within infected cells and eliminate the parasite. Consequently, dogs that relapsed had higher CIC levels, which may have been responsible for their inability to control the infection despite receiving a specific leishmanicidal treatment.

Importantly, our ROC curve analysis revealed that CIC were a good discriminator of relapse (AUC = 0.808) and that the optimal cut-off for accurately identifying relapse was 1.539 OD. This is relevant as the cut-off for positivity adopted in the study by Parody et al. (11) was 0.274 OD, which may be too low to be associated with clinical disease. In fact, CIC levels ranged between 0.8 and 1.0 OD in clinically healthy infected dogs, while sick dogs had levels between 1.9 and 3.1 OD. Further larger studies are needed to confirm our findings and establish definitive cut-offs to distinguish between healthy infected and sick dogs.

It is important to mention that our study did not include a formal sample size calculation due to the lack of prior data concerning expected effect sizes for the novel biomarker under investigation. However, our study yielded statistically significant results, providing valuable insights into the association between CIC clinical signs and clinicopathological parameters related to CanL. Moving forward, sample size calculations in future studies could enhance the robustness of our findings and contribute to a deeper understanding of CIC’s role in the progression of the disease.

## 5 Conclusion

Our findings indicate elevated serum CIC concentrations in both young adult dogs and/or those in advanced stages of CanL with anemia and/or dysproteinemia. Dogs showing a favorable response to treatment showed declining CIC levels. Conversely, dogs displaying a poor treatment response and recurrent clinical relapses consistently exhibited high CIC levels. In these dogs, vigilant monitoring is essential to enable prompt and targeted treatment and prevent irreversible organ damage due to CIC deposition.

While studies in larger populations of dogs are still needed to confirm our findings, the use of CICs as a complementary biomarker to track disease progression in CanL is promising. Further studies using this new biomarker may provide insight into specific organ tropism and relapse mechanisms in non-responders. Such studies will also improve our understanding of long-lasting cell-mediated immunity in resistant dogs, and aid in the development of *Leishmania* vaccines.

## Data availability statement

The raw data supporting the conclusions of this article will be made available by the authors, without undue reservation.

## Ethics statement

The animal studies were approved by Ethical committee Universidad Complutense de Madrid. The studies were conducted in accordance with the local legislation and institutional requirements. Written informed consent was obtained from the owners for the participation of their animals in this study.

## Author contributions

JS: Data curation, Formal analysis, Investigation, Methodology, Project administration, Resources, Writing – original draft, Writing – review & editing. NP: Investigation, Validation, Writing – review & editing. AM: Resources, Writing – review & editing. CC-L: Investigation, Validation, Writing – review & editing. JB: Resources, Writing – review & editing. RC: Resources, Writing – review & editing. MD: Resources, Writing – review & editing. JC: Conceptualization, Funding acquisition, Project administration, Supervision, Writing – review & editing. GM: Conceptualization, Funding acquisition, Methodology, Project administration, Resources, Supervision, Writing – review & editing.
