# Corrigendum: Clinical validation of circulating immune complexes for use as a diagnostic marker of canine leishmaniosis

**DOI:** 10.3389/fvets.2024.1423681

**Published:** 2024-05-13

**Authors:** Juliana Sarquis, Nuria Parody, Ana Montoya, Cristina Cacheiro-Llaguno, Juan Pedro Barrera, Rocío Checa, María Angeles Daza, Jerónimo Carnés, Guadalupe Miró

**Affiliations:** ^1^Department of Animal Health, Faculty of Veterinary, Universidad Complutense de Madrid, Madrid, Spain; ^2^R&D Unit Allergy and Immunology, LETI Pharma S.L.U., Madrid, Spain; ^3^Faculty of Veterinary, Small Animal Emergency and ICU Service, Veterinary Teaching Hospital, Universidad Complutense de Madrid, Madrid, Spain

**Keywords:** *Leishmania infantum*, immune complexes deposition, canine leishmaniosis, biomarker, PEG-ELISA

In the published article, there was an error. The symbol > is inverted.

A correction has been made to 3.3 CIC and laboratory findings, paragraph 7. This sentence previously stated:

“Dogs with borderline proteinuria (UPC = 0.2–0.5) had higher CIC levels than dogs without proteinuria (UPC > 0.5) (*p* = 0.035, r = 0.172).”

The corrected sentence appears below:

“Dogs with borderline proteinuria (UPC = 0.2–0.5) had higher CIC levels than dogs without proteinuria (UPC < 0.5) (*p* = 0.035, r = 0.172)”

The authors apologize for this error and state that this does not change the scientific conclusions of the article in any way. The original article has been updated.

